# A comparison of frequentist and Bayesian methods for meta-analysis of diagnostic test accuracy studies

**DOI:** 10.1017/S0950268826101551

**Published:** 2026-04-30

**Authors:** Xanthoula Rousou, Luis Furuya-Kanamori, Polychronis Kostoulas, Suhail A. R. Doi, Eleftherios Meletis, Nikolaos Solomakos

**Affiliations:** 1 Public and One Health, University of Thessaly, School of Health Sciences, Greece; 2 The University of Queensland Centre for Clinical Research, Australia; 3 Public and One Health, University of Thessaly, Greece; 4 Population Medicine, Qatar University College of Medicine, Doha, Qatar; 5 Veterinary Medicine, University of Thessaly School of Health Sciences, Greece

**Keywords:** Bayesian latent class, bivariate, *Campylobacter* spp, sensitivity, specificity, split component synthesis

## Abstract

Diagnostic test accuracy studies assess a diagnostic test’s performance against a reference standard. In this review, we explore and compare statistical methods used in meta-analyses of diagnostic test accuracy studies. Specifically, we evaluate two frequentist methods – split component synthesis (SCS) and bivariate model (BM) – alongside two Bayesian approaches: Bayesian hierarchical summary receiver operating characteristic (BHSROC) and Bayesian bivariate model (BBM). We also include their latent class variants (LC-BHSROC and LC-BBM). Using a meta-analysis of various multiplex nucleic acid amplification tests (NAATs/PCRs) against *Campylobacter* spp. *as a case study* we illustrate the practical applications of these methods. The reference standard was culture, and due to differences in cut-off values and primers among the NAAT/PCR brands, substantial heterogeneity was anticipated. Our findings reveal that the BM and BBM methods tend to estimate higher sensitivities than the other approaches, even when the number of studies is substantial, and heterogeneity is moderate – as observed in this case study. In such scenario, the SCS method or the BHSROC model may offer more robust and reliable outcomes. While our review is based on a real-life meta-analysis rather than simulations, it offers practical insights into the strengths and limitations of these statistical approaches for diagnostic test accuracy studies.

## Introduction

Meta-analysis of diagnostic test accuracy (DTA) studies constitutes a novel field of research, and a handful of methods address the intriguing nature of these studies [1–5]. The sensitivity (Se) and specificity (Sp) of a test define its accuracy, and owing to their dependent relation, their separate estimation is deemed unsound [2]. An older approach to their joint estimation was the Moses – Shapiro summary receiver operating characteristic (ROC) model [4], which returns a summary ROC curve and a summary estimate of the diagnostic odds ratio (DOR). The bivariate random effects method (linear mixed model) [2], which was subsequently updated via a generalized linear mixed model approach [6], generates paired Se and Sp estimates, and is currently the most routine method used for the meta-analysis of DTA studies [7]. This approach [8] is an extension of the generalized linear model (GLM) [9] that combines a GLM with normal random effects on the linear predictor scale. However, as illustrated by several authors [3, 10, 11], it underperforms when there is high heterogeneity and fewer studies. The split component synthesis (SCS) method has been suggested to be a better solution when heterogeneity across studies is high and overdispersion is present, which is commonly observed between DTA studies because of some degree of variation in test performance, depending on the tested patients’ characteristics such as disease severity, age, prevalence (spectrum effect) or on the cut-off values used by different studies (threshold effect) [3, 11] .

For Bayesian inference, two model types can be distinguished: (a) the Bayesian hierarchical summary receiver operating characteristic curve (BHSROC) model, first developed by Rutter and Gastonis [1] and updated by Dendukuri et al. [12], and (b) the Bayesian bivariate model (BBM) [5]. Both Bayesian models follow a hierarchical structure with between- and within-study levels, but only the BHSROC model accounts for threshold variability since it includes a threshold parameter in the model [1, 12]. The BBM is similar to the respective frequentist model [2] since both models incorporate a variance–covariance matrix for the between-study level to account for the correlation between Se and Sp [5].

All the above methods assume that the reference method’s outcome defines the true disease status (gold standard), but in some situations, the reference method provides imperfect information regarding the true disease status, it becomes unknown, hence latent. Bayesian latent class (LC) models presume the presence or absence of the disease as a dichotomous latent variable that is unobserved [1, 5, 12]. Therefore, these models calculate the probability of an individual having the true disease status and obtain the posterior distributions of the Se and Sp of both the index and the imperfect reference test, which amounts to the degree of belief in their true value, by combining prior information with the observed test outcomes [13]. Thus, the latent class models allow the accuracy of both the index and reference tests to be investigated [1, 5, 12]. Both Bayesian models described above can be run as latent class models, denoted as the LC-HSROC and LC-BBM models respectively.

The choice of an appropriate statistical pathway for DTA studies is not always clear and depends on several factors, such as the number of studies, the amount of heterogeneity (some due to spectrum effect), and whether a gold standard or latent class assumption is utilized. Therefore, the scope of this review paper is, via a case study, to compare and evaluate six methods: the two frequentist (SCS and BM) and the two Bayesian methodologies with (BHSROC and BBM) or without (LC-BHSROC and LC-BBM) the gold standard assumption.

## Methods

### Case study

Foodborne diseases are a significant public health challenge of high morbidity, with the global burden leading to a loss of 33 million healthy life years each year [14]. *Campylobacter* spp. constitute the most typical cause of foodborne illness and outbreaks in Europe and the United States [15, 16] and account for the loss of the equivalent of approximately 2.1 million years of full health (DALYs) annually [14].

Culture-independent tests (CIDTs) (antigen-based and molecular methods), such as singleplex or multiplex nucleic acid amplification tests (NAATs/PCRs), provide a quicker diagnosis than does culture [17], the reference method, and have excellent accuracy, as shown in our previous meta-analysis [18].

There is uncertainty around the clinical relevance of the multiplex NAAT/PCR’s excess positives. Positive NAAT/PCR results do not necessarily imply actual disease because these tests cannot provide information on whether it is an active infection, since no isolate can be provided [15, 19]. Therefore, the main question remains: when there is a negative culture and a positive NAAT/PCR result, is there a false positive result for multiplex NAAT/PCR or a true positive result due to the low Se of culture? Some studies reported culture Ses as low as 65% and 72% [20, 21], implying that culture is an imperfect reference standard. Therefore, the BLCM allows an evaluation of the accuracy of culture and NAAT/PCR as opposed to the frequentist methods that only offer results for the index test.

All multiplex NAATs/PCRs have primers that detect different parts of *Campylobacter jejuni* and *Campylobacter coli* [18]*;* only Luminex and Filmarray contain primers for *Campylobacter lari* and *C.upsaliensis* respectively [22]. Thus, if another Campylobacter species or subspecies is responsible for the infection, it is most likely that the culture method will detect it, and the index test’s outcome will be a false negative, as shown previously [23], where the index test Fast-Track Diagnostics could not identify the culture-isolated *C. lari.* In [17], the index test could not detect culture-isolated *C. hyointestinallis.* However, in [24], culture failed to isolate *C.upsaliensis*, probably due to the vulnerability of this particular strain to the antibiotics used in culture methods [25]. This case study therefore, provides an ideal setting for evaluating current frequentist and Bayesian methodologies since there are various index test brands with different primers, spectrum variations, a high number of included studies, and an imperfect reference standard.

#### Literature search

Data from a previous meta-analysis and from a new search extending from April 2021 to October 2022, which produced an additional five studies, yielding a total of 39 studies (Supplementary Figure S1). We searched Scopus, Science Direct, PubMed, Web of Science, and Mendeley, and the keywords used were: ‘Campylobacter’ AND ‘diagnostic’ AND ‘test’ AND ‘accuracy’ OR ‘Campylobacter’ AND ‘diagnostic’ AND ‘test’ AND ‘sensitivity’ OR ‘Campylobacter’ AND ‘diagnostic’ AND ‘test’ AND ‘specificity’. Studies were considered for inclusion if: (a) samples were collected, either prospectively or retrospectively, from patients with suspected gastro enteric infection, (b) multiplex NAT/PCR was the index test for the diagnosis of *Campylobacter* spp. (*C. jejuni* and *C. coli*), with simultaneous application of culture, the reference test, in every sample, (c) studies were peer-reviewed original articles, and (d) were written in English. A study was excluded if: (a) sampling of food, animals, or water, (b) evaluation of antimicrobial resistance tests and Campylobacter epidemiology, and (c) studies investigating *Helicobacter* spp. After the removal of duplicates, the first step was title and abstract screening and second full text evaluation, including reference lists from all included studies. The included studies evaluated the performance of multiplex NAATs/PCRs against culture, the reference standard. Some studies have evaluated the performance of more than one index test against cultures. The characteristics and the accuracy measures of the included studies, and for the newly included five studies in Supplementary Tables S1–S2. For more details, please refer to our previous paper [18].

Subgroup analysis per index test was performed to evaluate heterogeneity and the performance of the frequentist and Bayesian methods in different scenarios of heterogeneity levels and numbers of studies. The analysis accounted for only the index tests evaluated by ≥5 studies, which were Luminex, Filmarray, Allplex/Seeplex, and BDMax.

The index *I*^2^ was used to estimate the level of heterogeneity, which is incorporated within the function SCSMeta and the shiny apps. When the value is less than 25%, heterogeneity is considered low; when it is between 25% and 75%, heterogeneity is moderate; and when it is greater than 75%, heterogeneity is high.

### Frequentist methods

#### SCS method

The SCS method [3], a distributional-assumption free model, estimates the DOR from each study and combines the estimates across studies via the inverse variance heterogeneity (IVhet) meta-analytic model [26] and then splits the summary DOR from the meta-analysis via ordinary least squares (OLS) regression to its components Se and Sp. We performed the SCS method via the SCSMeta function [3] within the statistical package of the statistical software R [27].

#### Bivariate model

The frequentist BM uses a GLMM approach [2] and considers Se and Sp as a pair, particularly a vector, following a normal distribution, and derives the summary estimates via a mixed model approach assuming random effects for their often negative correlation. For this method, we utilized the shiny apps Meta-Disc 2.0 [28] and MetaDTA [29, 30]. The package mada [31] of the statistical software R [27] was not used because of inconsistent results.

### Bayesian methods

As previously described [1, 12], the BHSROC model accounts for variability and uncertainty in the data through a two-level hierarchical framework: (a) within-study level (parameters. 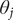 and 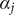 ) and (b) between-study level (hyperparameters 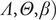 . The within-study parameter 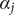 , accounts for diagnostic accuracy, and 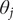 represents the threshold or cut-off value used to define a positive test result in that study, hence accounting for any differences in cut-off values across studies, which in our case study is the diversity in thresholds used across the different multiplex NAAT/PCR brands available. These within-study parameters are assumed to vary randomly across studies and are modelled using the between-study hyperparameters, where 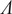 represents the average accuracy across studies, 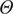 the average threshold and 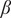 the scale parameter that provides the asymmetry and shape of the sROC curve.

The BM model adopts a direct modelling approach, treating the logit-transformed Se and Sp from each study as the primary outcomes. It assumes that the pair follows a bivariate normal distribution, characterized by a mean vector (representing the average logit of Se and logit of Sp across studies), a variance–covariance matrix, and a correlation coefficient r, which quantifies the potential trade-off between Se and Sp due to threshold effects or measurement variation.

In contradiction to the BHSROC model, the BM model does not explicitly model the threshold as a latent parameter. Instead, it implicitly assumes that all studies apply similar criteria to define a positive result or that the correlation term r captures any threshold-related heterogeneity. While both models ultimately provide estimates of pooled Se and Sp, the BHSROC model offers a more flexible framework for modelling threshold variation and generating a summary ROC curve, making it particularly suitable in situations where thresholds differ markedly across studies.

#### Prior distributions

We investigated the accuracy of both multiplex NAATs/PCRs and culture tests via weakly informative priors (restriction of the Se and Sp of culture to above 0.3 and 0.7 respectively) and informative priors for the Se and Sp of culture. All the prior distributions can be found in Tables 1 and 2. In addition, the informative prior beta distributions were calculated via the PriorGen package [32]. Two studies provided prior information concerning the accuracy of culture [20, 24]. However, since there were discrepancies regarding the Se of culture (reported as 60% and 72% respectively), three informative priors for Se were used to reflect this uncertainty. In contrast, as both studies reported Sp values above 90%, a single informative prior was used for Sp.

**Table 1.** Mean and range for the sensitivity (Se) and specificity (Sp) priors of culture and the corresponding beta distributions, beta (a, b)

Parameter	Mean	Range (min–max)	beta (a, b)
*Se*	0.8	0.66–0.9	*beta (126.13, 31.53)*
*Se*	0.75	0.62–0.86	*beta (142.57, 47.52)*
*Se*	0.6	0.28–0.88	*beta (15.79, 10.52)*
*Sp*	0.95	0.8–0.999	*beta (*63.2, 3.33*)*

**Table 2.** Prior distributions for the prevalence (pi), sensitivity (Se), and specificity (Sp) of multiplex NAAT/PCR (αi, θi, β, Λ, Θ, σ1, σ2)

Parameter	Hierarchical distribution	Prior distribution
p_i_		*beta (1,1)*
BHSROC		
α_i_	*~N (Λ, σ1)*	*Λ ~ U (−6, 6)* *s_1_ ~ dgamma (4*, *2)*
θ_i_	*~N (Θ, σ2)*	*Θ ~ U (−10, 10)* *s_2_ ~ dgamma (4*, *2)*
Β		*U (−2,2)*
Bivariate		
μ_se_	*~N(m_1_, σ_se_)*	*m_1_ ~ dnorm(0, 0.25)*
μ_sp_	*~N(m_2_, σ_sp_)*	*m_2_ ~ dnorm(0, 0.25)*
σ_se_, σ_sp_		*s_se_, s_sp_ ~ dgamma (2, 0.5)*
r		*r ~ U (−1, 1)*

#### Sensitivity analysis

Owing to the derivation of results from the same samples or the same biological process, the conditional dependence between the two tests, multiplex NAAT/PCR and culture, must be investigated to avoid biased estimations of the accuracy measures [5, 12]. Because the specificities for both tests are expected to be high, we only considered the conditional dependence of the sensitivities. However, in the case of the BHSROC model, it would be unidentifiable because the number of unknown parameters for each study exceeds the three degrees of freedom each study contributes to the model. Consequently, we investigated conditional dependence by considering the covariance between sensitivities to be fixed at 5%, 10%, 20%, 40%, or 50% of its maximum value, as illustrated previously [5].

To evaluate the effect of prior information on the posterior estimates, we ran the model with non-informative priors for Se and Sp of culture. We calculated the residual deviance (as explained previously [5]) and the deviance information criterion (DIC) module in rjags to assess model fit.

#### Convergence

Convergence was assessed by several diagnostic tools, such as sample effective size (SSeff > 400), autocorrelation (AC < 0.5), and Gelman and Rubin’s potential scale reduction factor (psrf < 1.05). In addition, convergence was visualized with the help of a series of plots, such as autocorrelation, trace, and density plots.

#### Software

We employed the runjag package [33] of the statistical software R to apply the Bayesian methods (model syntax code in the Supplementary Material). Two chains with different initial values were run, with 100000 iterations and a burn-in phase of 15000. We also performed the bivariate latent class model via the Shiny app bayesdta [34].

## Results

### Overall analysis

**Figure 1.**
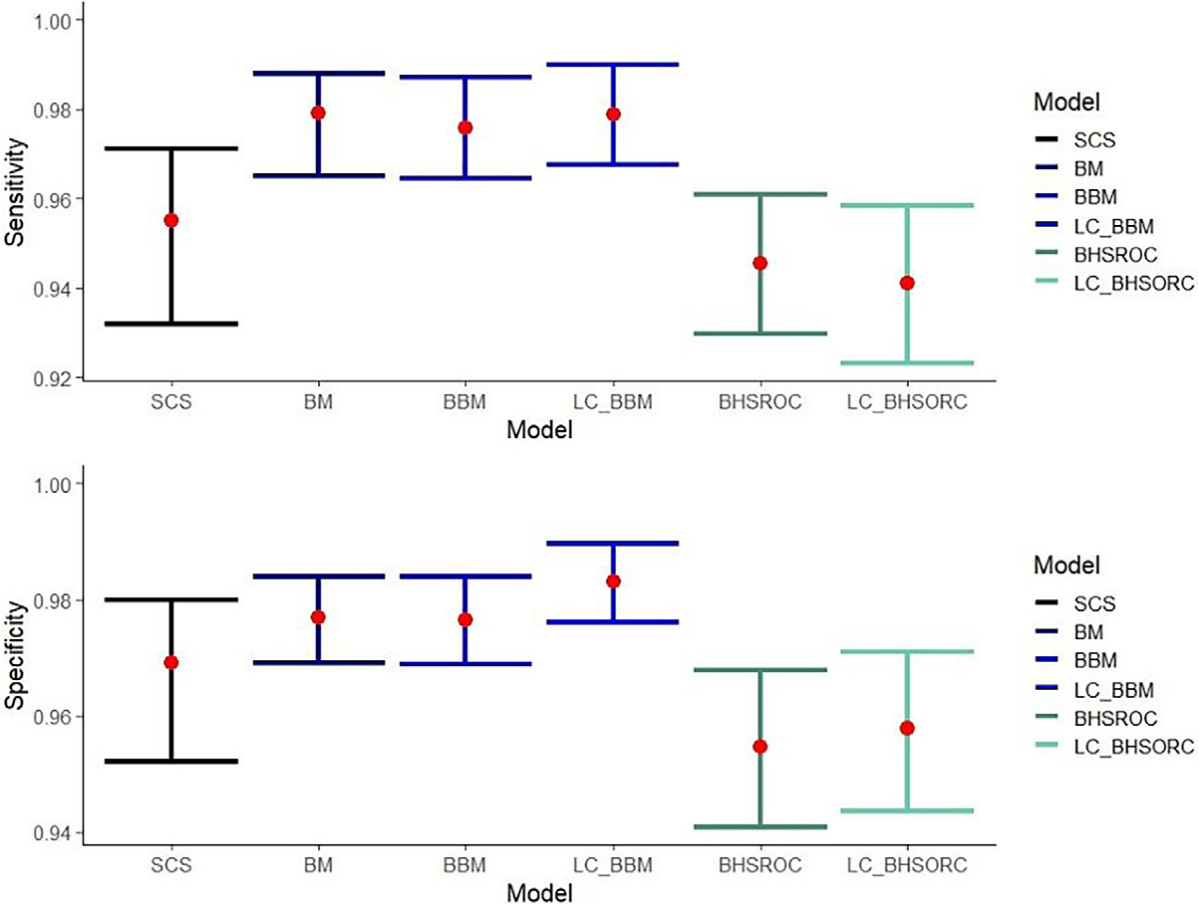
Sensitivity and specificity estimates with corresponding confidence/credible intervals (error bars) of multiplex NAAT/PCR against *Campylobacter* spp., compared to culture with or without the assumption of gold standard. Abbreviations: BBM, Bayesian bivariate model; BHSROC, Bayesian hierarchical summary receiver operating characteristic model; BM, Bivariate method; LC_BBM, Latent class Bayesian bivariate model; LC_BHSROC, Latent class Bayesian hierarchical summary receiver operating characteristic model; SCS, split component synthesis.

The multiplex NAAT/PCR accuracy measures, which are calculated via the different methods, are presented in Figure 1. The results from the LC-BHSROC model with the use of different priors and with model dependence can be found in Supplementary Tables S3 and S4, respectively.

The bivariate methods (BM and BBM) estimated a higher Se point estimate with narrower confidence/credible intervals than the SCS and BHSROC models. The BHSROC had the lowest Se estimate, with confidence/credible intervals not overlapping the corresponding intervals from the bivariate methods and wider than those of the SCS method.

The Sp values varied slightly among the different models, with the BHSROC model having the lowest estimate. Additionally, the corresponding credible interval was broader than those of the other methods. In the case of the BHSROC, there was overlap only with the respective intervals from the SCS method.

The pooled mean estimates of Se and Sp of the multiplex NAATs/PCRs, with the BHSROC, were 94.54 (95% CI: 92.98, 96.09) and 95.47 (95% CI: 94.09, 96.77) respectively. Furthermore, the latent class assumption resulted in only minor differences, suggesting that culture was close to a perfect gold standard. Indeed, the accuracy of culture was excellent and higher than that of the index test, with a Se of 97% and a Sp close to one (Table 3).

**Table 3.** Accuracy estimates with 95% credible intervals (95% CC) from the meta-analysis of the included studies with the Bayesian methods bivariate and the HSROC without the assumption of a gold standard

Accuracy estimates	LC-BHSROC Estimate % (95% CC)	LC-BBM Estimate % (95% CC)
Se index	94.1 (92.32,95.83)	97.89 (96.75,98.99)
Sp index	95.78 (94.35,97.09)	98.31 (97.6,98.96)
Se culture	97.09 (95.19,98.92)	96.69 (94.84,98.39)
Sp culture	99.98 (99.95,1)	99.98 (99.99,1)

### Subgroup analysis

**Figure 2.**
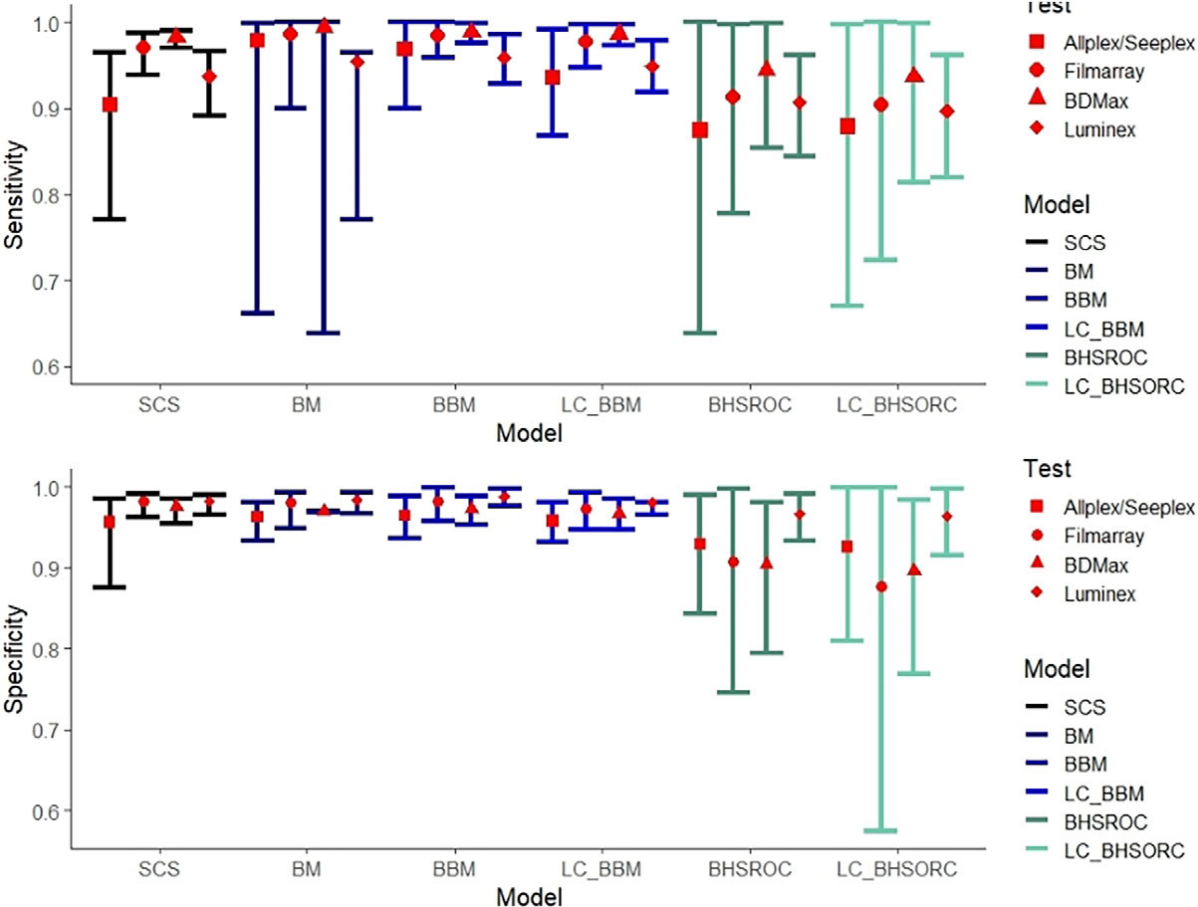
Sensitivity and specificity estimates with corresponding confidence/credible intervals (error bars) of index test Allplex/Seeplex, Filmarray, BDMax, and Luminex with number of studies 5, 5, 6(7 datasets), and 11(12 datasets), respectively. Heterogeneity (*I*^2^) for each subgroup was estimated 45.16%, 0%, 0%, and 62.48%, respectively. Abbreviations: BBM, Bayesian bivariate model; BHSROC, Bayesian hierarchical summary receiver operating characteristic model; BM, Bivariate method; LC_BBM, Latent class Bayesian bivariate model; LC_BHSROC, Latent class Bayesian hierarchical summary receiver operating characteristic model; SCS, split component synthesis.

In our study, the overall *I*^2^ was approximately 57%. Subgroup analysis revealed high heterogeneity for the Allplex/Seeplex and Luminex index tests and homogeneity in the Filmarray and BDMax subgroups. All methods had analogous estimates for the Filmarray and BDMax subgroups (Figure 2), except for the BHSROC, which produced the lowest estimates. The bivariate method showed higher Se values for the Luminex and Allplex/Seeplex subgroups than did the Bayesian HSROC and SCS methods, but the overlapping confidence/credible intervals suggest only numerical differences between the methods. The SCS and BHSROC methods’ estimates in the subgroup analysis were similar, with the SCS method estimating higher Se values and narrower confidence intervals overall (Figure 2).

### Sensitivity analysis-conditional dependence model

Including prior knowledge for the accuracy of culture in the model did not provide a better fit or influence the posterior distribution of the estimates, and there were minor differences in the pooled estimates between the models used (Supplementary Table S3). In the conditional dependence scenario, the pooled estimates were approximately the same as those in the conditional independence model and gradually decreased as the percentage increased (Supplementary Table S4).

## Discussion

DTA studies evaluate the performance of a diagnostic test against the reference standard. Therefore, the meta-analysis of DTA studies poses a challenge, particularly in the pairwise analysis of Se and Sp [2], which can be further complicated by a probable variation in the disease spectrum and threshold values. This review aimed to compare current and novel statistical approaches in a case study involving multiplex NAAT/PCRs for *Campylobacter* spp., with implicit threshold variations and differing primers across various test brands.

Our findings showed that, when using the BHSROC model with a gold standard, the estimated Se and Sp of multiplex NAAT/PCR were 94.54 (95% CI: 92.98, 96.09) and 95.47 (95% CI: 94.09, 96.77) respectively. The inclusion of the new studies did not influence the Se of multiplex NAAT/PCR, but decreased the Sp by almost 1%. The Se and Sp were 95.3% (92.3; 97.1) and 97.1% (95.1; 98.3). Minor differences were observed when assuming an imperfect gold standard, perhaps due to the excellent Sp and Se of culture.

Notably, our study revealed that culture had a much higher Se of 97% (95% CI: 95.2, 98.96) than previously reported in other studies [20–22]. This contrast may be partly explained by the composite reference tests used in previous evaluations, which often relied on other multiplex or singleplex NAAT/PCR assays to adjudicate discordant results. Since these NAAT/PCRs may have lower Sp, particularly due to the detection of non-viable organisms, it is plausible that the observed discordance was driven by false positives from NAAT/PCR rather than false negatives from culture. Consequently, prior estimates may have underestimated the true Se of culture. Furthermore, we tested for conditional dependence between culture and the index test, which revealed only minor differences in the posterior estimates for both tests, suggesting that the latent class model’s assumption of conditional independence did not substantially inflate the estimated test accuracies. While culture and NAAT/PCR showed high concordance – particularly for positive results – the stability of estimates under conditional dependence modelling supports the robustness of our findings. The results from the BM and BBM models yielded higher Se point estimates for the index test, consistent with findings from previous studies [10, 11] evaluating the performance of the bivariate model. However, Sps were similar in all methods except for the BHSROC. Furthermore, the confidence/credible intervals were broader for the Bayesian models and the SCS method compared to the BM, which probably suffers from overdispersion due to its reliance on the random effects assumption [35]. In scenarios where moderate heterogeneity is observed – primarily reflecting spectrum effects and variations in cutoff values – the BM and BBM may provide different estimates than the SCS and BHSROC methods.

Three factors are considered critical for the performance of the BM: the number of studies, the total study population, and heterogeneity [10]. In contrast, both the SCS and BHSROC methods have been found to be more robust than the BM in the presence of high heterogeneity (more than 80%) and a limited number of studies [11]. Although our study included a large number of studies and sample sizes, and heterogeneity was moderate, the BM still produced slightly different accuracy estimates than the BHSROC and SCS methods.

In subgroup analyses where within-subgroup heterogeneity was absent, the frequentist and Bayesian methods (except for BHSROC) provided comparable estimates. However, in subgroups with moderate to high within-subgroup heterogeneity, such as the Allplex/Seeplex and Luminex subgroups, the BM/BBM methods yielded numerically higher Se estimates than the SCS and BHSROC methods. This discrepancy could be explained by the variability in the strains isolated and the primers used by each test, potentially influencing the observed heterogeneity in these subgroups. The amplified Se estimates from the BM models might reflect these differences in strains or diagnostic thresholds. In this context, the BHSROC and SCS methods may be more reliable for providing consistent estimates, particularly when the heterogeneity is driven by variations in *Campylobacter* spp. or isolated strains [3, 12].

In conclusion, BM seems to overestimate the Se compared with the other methods, even when the number of studies is noteworthy, and heterogeneity is moderate, as demonstrated in our case study. This overestimation may stem from the BM’s handling of implicit threshold differences. Therefore, for more robust outcomes, we recommend the use of the SCS method or the BHSROC model, especially in cases with moderate to high heterogeneity. However, as our comparison was based solely on real-world data, further research using simulations with different scenarios is needed to further refine and validate these methodologies, ensuring their broader applicability in diagnostic test accuracy evaluations.

## Supporting information

10.1017/S0950268826101551.sm001 Rousou et al. supplementary material Rousou et al. supplementary material

## Data Availability

All data generated or analysed during this study are included in this published article (and its Supplementary Information files).

## Supplementary material

The supplementary material for this article can be found at http://doi.org/10.1017/S0950268826101551.
