# Supplementary material for: A comparison of frequentist and Bayesian methods for meta-analysis of diagnostic test accuracy studies
Source: Epidemiol Infect. 2026 Apr 30;154:e61. doi: 10.1017/S0950268826101551 (PMC13184654; doi:10.1017/S0950268826101551)
Supplement: Rousou et al. supplementary material [file S0950268826101551sup001.docx]

# Supplementary material

[Table S1.Characteristics of included studies concerning country, study design (retrospective or prospective), participants’ characteristics (age, symptoms), Campylobacter species tested by the index test, funding or not by the index test’s manufacturer and culture method information (transport medium used, agar, identification method). Blank cells in the table depict no information is provided by the authors of the study.](#_Toc203559453)

[Table S2. Accuracy measures of included studies](#_Toc203559454)

[Table S3. Results from the Bayesian hierarchical latent class (LC-BHSROC) model](#_Toc203559455)

[Table S4. Results from the conditional dependence Bayesian hierarchical latent (LC-BHSROC) model with weakly informative priors for the reference test.](#_Toc203559456)

[Figure S1: The PRISMA flow diagram of the systematic process for the selection of the relevant studies^6^](file:///C:\Users\user\Desktop\Supplementary%20material_CAMP22.docx#_Toc164154583)

[**1 Model code for rjags-Bayesian HSROC**](#_Toc167277658)

[**2. Model code for rjags-Bayesian bivariate**](#_Toc167277659)

Table S1.Characteristics of included studies concerning country, study design (retrospective or prospective), participants’ characteristics (age, symptoms), Campylobacter species tested by the index test, funding or not by the index test’s manufacturer and culture method information (transport medium used, agar, identification method). Blank cells in the table depict no information is provided by the authors of the study.

| **Index test** | **Ntotal^1^** | **Country** | **Study design** | **Age** | **Samples** | ***Camp.* spp.**  **(index test)** | **Funded** | **Transport media** | **Culture information** | **Identification method** | **Study** |
| --- | --- | --- | --- | --- | --- | --- | --- | --- | --- | --- | --- |
| Allplex | 394 | Spain | prospective  (94 characterized) | paediatric and adult | diarrheal  faecal samples |  | Yes |  | Campylobacter agar 42°C | MALDI-TOF | Martin A. et al 2018 (1) |
| Allplex | 184 | Korea | prospective | pediatric |  |  |  | Cary-Blair transport medium | Campylobacter CVA agar, 42°C | MALDI-TOF | Jo S.J. et al. 2022 (2) |
| Amplidiag | 1168 | Sweden and Finland |  | paediatric and adult | faecal samples , routine testing | *C. jejuni, C.coli* | Yes |  |  |  | Rintala A. et al 2016  (3) |
| BDMax | 299 | Singapore | prospective | adult |  | C. jejuni and C.coli | No | No | Campylobacter selective agar, appropriate atmosphere, and conditions | MALDI-TOF | Koo S.H. et al. (4) |
| BDMax | 400 | Turkey | prospective | pediatric and adult |  | C. jejuni and C.coli |  | Cary-Blair transport medium | modified charcoal cefoperazone-deoxycholate agar, 42°C sub-cultured to 5% sheep blood agar medium and incubated for 48 h | mass spectometer, MALDI-TOF | Ozcan N. et al. (5) |
| BDMax | 161 | Canada | prospective |  |  | C. jejuni and C.coli | Yes, research grant external to the study | Cary-Blair transport medium | standard methodology | mass spectometer | Berenger B.M. et al. (6) |
| BD Max | 1056 | Switzerland | prospective |  |  | *C. jejuni, C.coli* | No | Cary-Blair for some samples | Campylosel agar 42°C | MALDI-TOF | Wohlwend N. et al 2016 (7) |
| BD Max | 2893 | USA, Canada, Mexico | prospective | paediatric and adult | soft or diarrheal faecal samples | *C. jejuni, C.coli* | Yes | Cary-Blair or dry clean container | selective campylobacter agar | hippurate/indoxyl acetate/16SrRNA sequence | Harrington et al 2015 (8) |
| BD Max | 507 | USA, Canada, Mexico | retrospective | paediatric and adult | soft or diarrheal faecal samples | *C. jejuni, C.coli* | Yes |  | selective campylobacter agar 42°C | hippurate/indoxyl acetate/16SrRNA sequence | Harrington et al 2015 (8) |
| BD Max | 893 | Austria | prospective |  | diarrheal faecal samples | *C. jejuni, C.coli* | No |  | specific Campylobacter medium at 37 °C overnight and 42°C for 2 days | MALDI-TOF | Knabl L. et al 2016  (9) |
| EntericBio | 237 | New Zealand | prospective | paediatric and adult | faecal samples , routine testing |  | No | No | Campylobacter blood free agar 42°C | MALDI-TOF | McAuliffe G.N. et al 2017 (10) |
| EntericBio | 773 | Ireland | prospective |  | symptoms of gastroenteritis |  | No |  | Preston agar 42°C | microscopic examination/catalase/oxidase | O’Leary J. et al 2009 (11) |
| EntericBio | 400 | Turkey | prospective | pediatric and adult |  | C. jejuni and C.coli |  | Cary-Blair transport medium | modified charcoal cefoperazone deoxycholate agar, 42°C sub-cultured to 5% sheep blood agar medium and incubated for 48 h | mass spectometer, MALDI-TOF | Ozcan N. et al.(5) |
| Fast-Track Diagnostics | 1758 | New Zealand | prospective | paediatric and adult | diarrheal  faecal samples | *C. jejuni, C.coli and C. lari* | Yes | No | Campylobacter blood free agar 42°C | MALDI-TOF | McAuliffe G.N. et al 2013 (12) |
| Fast track diagnostics | 276 | India | prospective | pediatric and adult |  | C. jejuni and C.coli and C.lari | No | No | Campylobacter selective agar, enrichment, standard methods |  | Ghoshal U. et al. (13) |
| Fast track diagnostics | 161 | Canada | prospective |  |  | C. jejuni and C.coli | Yes, research grant external to the study | Cary-Blair transport medium | standard methodology | mass spectometer | Berenger B.M. et al. (6) |
| Filmarray | 184 | Korea | prospective | pediatric |  | C. jejuni, C.coli and C. upsaliensis |  | Cary-Blair transport medium | Campylobacter CVA agar, 42°C | MALDI-TOF | Jo S.J. et al. 2022 (2) |
| FilmArray | 1556 | USA | prospective | paediatric and adult | faecal samples , routine testing | *C. jejuni, C.coli and C. upsaliensis* | Yes | Cary-Blair | Campylobacter CVA agar 42°C | standard procedures | Buss S.N. et al 2015(14) |
| FilmArray | 1887 | USA | prospective | paediatric and adult | symptoms of gastroenteritis | *C. jejuni, C.coli and C. upsaliensis* | Yes  test kits and instruments | Cary-Blair | Campylobacter selective agars 42°C for 3 days |  | Cybulski R.J. et al 2018 (15) |
| FilmArray | 152 | USA | prospective and retrospective | paediatric | acute gastroenteritis | *C. jejuni, C.coli and C. upsaliensis* | No | Cary-Blair | Campylobacter blood agar 42°C | hippurate test | Huang R.S.P. et al 2016 (16) |
| FilmArray | 106 | Japan | prospective | adult | travellers΄ diarrhea | *C. jejuni, C.coli and C. lari* | Yes  ICMJE form ^2^  machine and panel | No | Skirrow campylobacter selective agar | biochemical and phenotypic analyses | Kutsuna S. et al 2021 (17) |
| LD | 393 | Germany | prospective |  | diarrheal faecal samples | *C. jejuni* | No | No | Karmali agar 42°C | MALDI-TOF, hippurate | Wiemer D. et al 2011(18) |
| LD | 350 | Egypt | prospective | adult | symptoms of gastroenteritis | *C. jejuni, C.coli* |  | No | Skirrow's medium 37 °C | oxidase, gram stain, hippurate test | Zaghloul M.Z. et al 2012 (19) |
| LD | 1728 | China | prospective |  | diarrheal faecal samples | *C. jejuni* | No |  | enrichment broth, no info about type of agar, based on guidelines | biochemical and serological tests | Hu Q. et al 2014 (20) |
| LD (RFBS24 Qkit) | 47 | Japan |  |  | foodborne outbreaks | *C. jejuni, C.coli* | No |  |  |  | Kawase J. et al 2016 (21) |
| LD (RFBS24 Ukit) | 47 | Japan |  |  | foodborne outbreaks | *C. jejuni, C.coli* | No |  |  |  | Kawase J. et al 2016  (21) |
| LD (RFBS24 ver5 Qkit) | 47 | Japan |  |  | foodborne outbreaks | *C. jejuni, C.coli* | No |  |  |  | Kawase J. et al 2016  (21) |
| LD (RFBS24 ver5 Ukit) | 47 | Japan |  |  | foodborne outbreaks | *C. jejuni, C.coli* | No |  |  |  | Kawase J. et al 2016  (21) |
| Luminex | 3089 | Canada | prospective | paediatric | ≥3 episodes of vomiting and/or diarrhea in the preceding 24 h and <7 days of symptoms | *C. jejuni, C.coli and C. lari* | Yes | Cary-Blair | Campylobacter blood-free selective agar 42°C | MALDI-TOF | Kellner T. et al 2019(22) |
| Luminex | 254 | USA | prospective | paediatric and adult | symptoms of gastroenteritis | *C. jejuni, C.coli and C. lari* | No | Cary-Blair or in sterile cups | Campylobacter agar 42°C | Gram stain; catalase, oxidase, hippurate, indoxyl acetate, MIDI, MicroSEQ (16S sequencing) | Navidad J.F. et al 2013 (23) |
| Luminex | 290 | Southern China | prospective | paediatric and adult | diarrheal faecal samples | *C. jejuni, C.coli and C. lari* | No | No | Skirrow agar plates 42°C | MALDI-TOF | Deng J. et al 2015 (24) |
| Luminex | 479 | Vietnam |  | paediatric and adult | diarrheal faecal samples | *C. jejuni, C.coli and C. lari* | No | No | Campylobacter plates 42°C | hippurate test | Duong V.T. et al 2016 (25) |
| Luminex | 1396 | UK | prospective | paediatric and adult | diarrheal faecal samples | *C. jejuni, C.coli and C. lari* | Yes  ICMJE form ^2^  contribution to costs of consumables | No | Charcoal Cefoperazone Desoxycholate | biochemical identification, MALTI-TOF and/or basic serotyping | Halligan E. et al 2014 (26) |
| Luminex | 178 | Belgium | prospective | paediatric | acute gastroenteritis | *C. jejuni, C.coli and C. lari* | No | No | Butzler medium and a Columbia agar for the filtration method | MALDI-TOF | Tilmanne A. et al 2019 (27) |
| Luminex | 511 | canada,usa,uk, netherlands | prospective | paediatric and adult | faecal samples, routine testing | *C. jejuni, C.coli and C. lari* | Yes |  | standard procedures at all sites | standard procedures at all sites | Claas et al 2013(28) |
| Luminex | 217 | Taiwan | prospective | paediatric and adult | symptoms of gastroenteritis | *C. jejuni, C.coli and C. lari* | No |  | Campylobacter isolated agar | biochemical tests | Huang Shu-Huan et al 2018  (29) |
| Luminex | 937 | UK | retrospective | paediatric and adult | symptoms of gastroenteritis | *C. jejuni, C.coli and C. lari* | No |  | Campylobacter blood free agar 42°C, first selenine broth | identified to species level, no information to the technique | Pankhurst L. et al 2014 (30) |
| Luminex | 839 | UK | retrospective | paediatric and adult | symptoms of gastroenteritis | *C. jejuni, C.coli and C. lari* | No |  | Campylobacter blood free agar 42°C, first selenine broth | identified to species level, no information to the technique | Pankhurst L. et al 2014 (30) |
| Luminex | 211 | USA | prospective | paediatric and adult | symptoms of gastroenteritis | *C. jejuni, C.coli and C. lari* | No |  | Campylosel agar 42°C | catalase, oxidase, hippurate, indoxyl acetate, MIDI, MicroSEQ | Patel A. et al 2014  (31) |
| Luminex | 991 | UK | retrospective | paediatric and adult | diarrheal faecal samples | *C. jejuni, C.coli and C. lari* | Yes |  |  |  | Perry M.D. et al 2014 (32) |
| MassCode | 948 | UK | retrospective | paediatric and adult | symptoms of gastroenteritis | *C. jejuni and C.coli* | No |  | Campylobacter blood free agar 42°C, first selenine broth | identified to species level, no information to the techique | Pankhurst L. et al 2014 (30) |
| NanoCHIP1 | 94 | Israel | prospective |  | symptoms of gastroenteritis | *C. jejuni and C.coli* | No | Cary-Blair | Campylobacter blood agar | standard procedures | Dror S.K. et al 2016  (33) |
| NanoCHIP1 | 161 | Israel | retrospective |  | frozen stool bank | *C. jejuni and C.coli* | No |  | Campylobacter blood agar | standard procedures | Dror S.K. et al 2016 |
| Novodiag | 251 | France | prospective and retrospective | paediatric and adult | No information | *C. jejuni and C.coli* | No, however kits provided by manufacturer | Cary-Blair for some samples | Campylosel agar | MALDI-TOF | Roy C. et al 2020  (34) |
| ProGastro | 161 | Canada | prospective |  |  | C. jejuni and C.coli | Yes, research grant external to the study | Cary-Blair transport medium | standard methodology | mass spectometer | Berenger B.M. et al. (6) |
| ProGastro | 105 | USA | retrospective | paediatric and adult | faecal samples , routine testing | *C. jejuni and C.coli* | Yes | Cary-Blair | Campylobacter blood agar 42°C | hippurate test | Buchan B.W. et al 2013(35) |
| ProGastro | 1139 | USA | prospective | paediatric and adult | faecal samples , routine testing | *C. jejuni and C.coli* | Yes | Cary-Blair | Campylobacter blood agar 42°C | hippurate test | Buchan B.W. et al 2013(35) |
| Savyon | 991 | UK | retrospective | paediatric and adult | diarrheal faecal samples | *C. jejuni and C.coli* | Yes |  |  |  | Perry M.D. et al 2014 (32) |
| Seeplex | 201 | UK | retrospective | paediatric and adult | faecal samples , no information | *C. jejuni and C.coli* | No | No | modified cefeoperazone, charcoal deoxycholate (CCD) agar, 37°C |  | Coupland L.J. et al 2013(36) |
| Seeplex | 242 | France | prospective | paediatric and adult | acute gastroenteritis | *C. jejuni and C.coli* |  | No | two culture methods: 1)filtration step,brucella broth/trypticase soy blood agar 37 °C  2) without filtration ,karmali agar 37 °C | motility, Gram staining, and  oxidase activity hippurate test | Bessede E. et al 2011(37) |
| Seeplex | 245 | Italy | prospective | paediatric and adult | diarrheal faecal samples |  | No |  | Campylosel agar 42°C | API-Campy system | Onori M. et al 2014 (38) |
| Verigene | 268 | Japan | prospective |  | faecal samples , routine testing | *C. jejuni, C.coli and C. lari* |  |  | Modified skirrow agar or mod campylobacter agar 10%SB | Api-Campy system or MALDI-TOF | Kosai K. et al 2021(39) |

^1^ Ntotal (total patient population sampled)

^2^ ICMJE (International Committee of Medical Journal Editors) form for Disclosure of Potential Conflicts of Interest

Table S2. Accuracy measures of included studies

| **Index test** | **Ntotal**^1^ | **TP**^1^ | **FP**^1^ | **TN**^1^ | **FN**^1^ | **Se**^1^ | **95%CI**^1^ | **Sp**^1^ | **95% CI**^1^ | **Study** |
| --- | --- | --- | --- | --- | --- | --- | --- | --- | --- | --- |
| Allplex | 394 | 21 | 23 | 350 | 0 | 100 | 84.54-100 | 93.83 | 90.92-95.86 | Martín A. et al. 2018(1) |
| Allplex | 184 | 17 | 3 | 164 | 0 | 100 | 81.57,100 | 98.2 | 94.85,99.39 | Jo S.J. et al. 2022 (2) |
| Amplidiag | 1168 | 57 | 27 | 1084 | 0 | 100 | 93.69-100 | 97.57 | 96.49-98.32 | Rintala A. et al. 2016(3) |
| BDMax | 161 | 115 | 6 | 40 | 0 | 100 | 96.77,100 | 86.96 | 74.33,93.88 | Berenger B.M. et al. (6) |
| BDMax | 299 | 18 | 15 | 266 | 0 | 100 | 82.41,100 | 94.7 | 91.38,96.74 | Koo S.H. et al. (4) |
| BDMax | 400 | 21 | 22 | 359 | 0 | 100 | (84.54,100 | 94.23 | 91.41,96.16 | Ozcan N. et al. (5) |
| BDMax | 2893 | 44 | 51 | 2797 | 1 | 97.78 | 88.43-99.61 | 98.21 | 97.65-98.64 | Harrington et al. 2015(8) |
| BDMax | 507 | 129 | 2 | 372 | 4 | 96.99 | 92.52-98.82 | 99.47 | 98.07-99.85 | Harrington et al. 2015(8) |
| BDMax | 893 | 28 | 13 | 851 | 1 | 96.55 | 82.82-99.39 | 98.50 | 97.44-99.12 | (Knabl L. et al. 2016(9) |
| BDMax | 1056 | 75 | 34 | 947 | 0 | 100 | 95.13-100 | 96.53 | 95.2-97.51 | Wohlwend N. et al. 2016(7) |
| EntericBio | 400 | 21 | 21 | 359 | 0 | 100 | 84.54,100 | 94.47 | 91.7,96.36 | Ozcan N. et al. (5) |
| EntericBio | 237 | 13 | 2 | 222 | 0 | 100 | 77.19-100 | 99.11 | 96.8-99.75 | McAuliffe G. et al. 2017(10) |
| EntericBio | 773 | 30 | 12 | 731 | 0 | 100 | 88.65-100 | 98.38 | 97.2-99.07 | O’Leary J. et al. 2009(11) |
| Fast-Track  Diagnostics | 1758 | 81 | 4 | 1670 | 3 | 96.43 | 90.02-98.78 | 99.76 | 99.39-99.91 | McAuliffe G.N et al.2013 (12) |
| Fast track diagnostics | 161 | 114 | 4 | 42 | 1 | 99.13 | 95.24,99.85 | 91.3 | 79.68,96.57 | Berenger B.M. et al. (6) |
| Fast track diagnostics | 276 | 0 | 4 | 272 | 0 | NA |  | 98.55 | 96.33,99.43 | Ghoshal U. et al. (13) |
| Filmarray | 184 | 18 | 2 | 164 | 0 | 100 | 82.41,100 | 98.795 | 95.71,99.67 | Jo S.J. et al. 2022 (2) |
| FilmArray | 1556 | 34 | 24 | 1497 | 1 | 97.14 | 85.47-99.49 | 98.42 | 97.66-98.94 | Buss S.N. et al. 2015(14) |
| FilmArray | 1887 | 50 | 17 | 1819 | 1 | 98.04 | 89.7-99.65 | 99.07 | 98.52-99.42 | Cybulski R.J. et al. 2018(15) |
| FilmArray | 152 | 11 | 1 | 140 | 0 | 100.00 | 74.12-100 | 99.29 | 96.09-99.87 | Huang R. S. P. et al. 2016(16) |
| FilmArray | 106 | 12 | 13 | 81 | 0 | 100.00 | 75.75-100 | 86.17 | 77.77-91.74 | Kutsuna S. et al. 2021(17) |
| Luminex | 511 | 111 | 15 | 382 | 3 | 97.37 | 92.55-99.1 | 96.22 | 93.86-97.7 | Claas et al. 2013(28) |
| Luminex | 290 | 20 | 16 | 254 | 0 | 100.00 | 83.89-100 | 94.07 | 90.59-96.32 | Deng J. et al. 2015(24) |
| Luminex | 479 | 27 | 35 | 414 | 3 | 90.00 | 74.38-96.54 | 92.20 | 89.35-94.34 | Duong V.T. et al. 2016 (25) |
| Luminex | 1396 | 23 | 37 | 1336 | 0 | 100.00 | 86.59-100 | 97.31 | 96.31-98.04 | Halligan E. et al. 2014 (26) |
| Luminex | 217 | 13 | 5 | 199 | 0 | 100.00 | 77.19-100 | 97.55 | 96.8-99.75 | Huang S. H. et al. 2018 (29) |
| Luminex | 3089 | 20 | 2 | 3064 | 3 | 86.96 | 67.87-95.46 | 99.93 | 99.76-99.98 | Kellner T. et al. 2019 (22) |
| Luminex | 254 | 18 | 1 | 233 | 2 | 90.00 | 69.9-97.21 | 99.57 | 97.62-99.92 | Navidad J.F. et al. 2013 (23) |
| Luminex | 937 | 197 | 14 | 724 | 2 | 98.99 | 96.41-99.72 | 98.10 | 96.84-98.87 | Pankhurst L. et al. 2014(30) |
| Luminex | 839 | 110 | 11 | 711 | 7 | 94.02 | 88.16-97.07 | 98.48 | 97.29-99.15 | Pankhurst L. et al. 2014 (30) |
| Luminex | 211 | 1 | 0 | 210 | 0 | 100.00 | 20.65-100 | 100.00 | 98.2-1 | Patel A. et al. 2014 (31) |
| Luminex | 991 | 42 | 30 | 917 | 2 | 95.45 | 84.87-98.74 | 96.83 | 95.51-97.77 | Perry M.D. et al 2014 (32) |
| Luminex | 178 | 19 | 3 | 153 | 6 | 76 | 56.57-88.5 | 98.08 | 94.5-99.34 | Tilmanne et al. 2020 (27) |
| MassCode | 948 | 181 | 40 | 708 | 19 | 90.50 | 86.54-93.83 | 94.65 | 92.8-96.05 | Pankhurst et al. 2014 (30) |
| LD | 1728 | 11 | 5 | 1717 | 0 | 100.00 | 74.12-100 | 99.71 | 99.3-99.88 | Hu et al. 2014 (20) |
| LD | 393 | 79 | 5 | 307 | 2 | 97.53 | 91.44-99.32 | 98.40 | 96.3-99.31 | Wiemer et al. 201 (18) |
| LD | 350 | 16 | 7 | 327 | 0 | 100.00 | 80.64-100 | 97.90 | 95.74-98.98 | Zaghloul et al. 2012 (19) |
| NanoCHIP1 | 94 | 3 | 4 | 87 | 0 | 100.00 | 43.85-100 | 95.60 | 89.24-98.28 | Dror K. et al. 2016 (33) |
| NanoCHIP1 | 161 | 26 | 1 | 133 | 1 | 96.30 | 81.72-99.34 | 99.25 | 95.89-99.87 | Dror K. et al. 2016 (33) |
| Novodiag | 251 | 61 | 31 | 159 | 0 | 100.00 | 94.08-100 | 83.68 | 77.77-88.26 | Roy C. et al. 2020 (34) |
| ProGastro | 161 | 111 | 4 | 42 | 4 | 96.52 | 91.4,98.64 | 91.3 | 79.68,96.57 | Berenger, B. M. et al.(6) |
| ProGastro | 105 | 27 | 5 | 72 | 1 | 96.43 | 82.29-99.37 | 93.51 | 85.68-97.19 | Buchan B.W. et al. 2013 (35) |
| NanoCHIP1 | 1139 | 20 | 13 | 1106 | 0 | 100.00 | 83.89-100 | 98.84 | 98.02-99.32 | Buchan B.W. et al. 2013 (35) |
| LD (RFBS24 Qkit) | 47 | 24 | 0 | 16 | 7 | 77.42 | 60.19-88.6 | 100.00 | 80.64-100 | Kawase J. et al. 2016 (21) |
| LD (RFBS24 Ukit) | 47 | 26 | 0 | 16 | 5 | 83.87 | 67.37-92.91 | 100.00 | 80.64-100 | Kawase J. et al. 2016 (21) |
| LD (RFBS24 ver5 Qkit) | 47 | 29 | 2 | 15 | 1 | 96.67 | 83.3-99.41 | 88.24 | 65.66-96.71 | Kawase et al. J. 2016 (21) |
| LD (RFBS24 ver5 Ukit) | 47 | 31 | 4 | 12 | 0 | 100.00 | 88.97-100 | 75.00 | 50.5-89.82 | Kawase J. et al. J. 2016 (21) |
| Savyon | 991 | 44 | 38 | 909 | 0 | 100.00 | 91.97-100 | 95.99 | 94.54-97.06 | Perry M.D. et al. 2014 (32) |
| Seeplex | 242 | 13 | 10 | 216 | 3 | 81.25 | 56.99-93.41 | 95.58 | 92.05-97.58 | Bessède E. et al. 2011 (37) |
| Seeplex | 201 | 16 | 2 | 183 | 0 | 100.00 | 80.64-100 | 98.92 | 96.14-99.7 | Coupland L.J et al. 2012 (36) |
| Seeplex | 245 | 4 | 15 | 223 | 1 | 80.00 | 37.55-96.38 | 93.70 | 89.86-96.14 | Onori M.et al. 2014 (38) |
| Verigene | 268 | 47 | 2 | 219 | 0 | 100.00 | 92.44-100 | 99.10 | 96.76-99.75 | Kosai K. et al. 2021 (39) |

^1^ Ntotal (total patient population sampled), TP (True Positive), FP (False Positive), TN (True Negative), FN (False Negative), Se (Sensitivity), Sp (Specificity), 95%CI (95 % Confidence Interval)

Table S3. Results from the Bayesian hierarchical latent class (LC-BHSROC) model

| **Parameter Estimates** | **Model- Conditional independence** |
| --- | --- |
|  | **mean % (95%CI)** |
| ***Non-informative priors*** |  |
| Sensitivity (*Multiplex PCR*) | 94.09 (92.23,95.81) |
| Specificity (*Multiplex PCR)* | 95.78 (94.34, 97.08) |
| Sensitivity *(culture)* | 97.1 (95.2,98.96) |
| Specificity *(culture)* | 99.98 (99.96,1) |
| ***Restricted priors for reference test*** |  |
| Sensitivity (*Multiplex PCR*) | 94.1 (92.32,95.83) |
| Specificity (*Multiplex PCR*) | 95.78 (94.35,97.09) |
| Sensitivity *(culture)* | 97.09 (95.19,98.92) |
| Specificity *(culture)* | 99,98(99.99,1) |
| ***Informative prior for the sensitivity of culture, beta (126.13,31.53)*** |  |
| Sensitivity (*Multiplex PCR*) | 93.5 (91.52,95.4) |
| Specificity (*Multiplex PCR*) | 96.19 (94.75,97.46) |
| Sensitivity *(culture)* | 93.63 (92.12,95.14) |
| Specificity *(culture)* | 99,98 (99.96,1) |
| ***Informative prior for the sensitivity of culture, beta (142.57,47.52)*** |  |
| Sensitivity (*Multiplex PCR*) | 93.34 (91.25,95.21) |
| Specificity (*Multiplex PCR*) | 96.3 (94.87,97.63) |
| Sensitivity *(culture)* | 92.35 (90.77,93.83) |
| Specificity *(culture)* | 99,98 (99.96,1) |
| ***Informative prior for the sensitivity of culture, beta (15.79,10.52)*** |  |
| Sensitivity (*Multiplex PCR*) | 94.02 (92.15,95.79) |
| Specificity (*Multiplex PCR*) | 95.8 (94.33,97.18) |
| Sensitivity *(culture)* | 95.27 (93.64,96.85) |
| Specificity *(culture)* | 99,96 (99.96,1) |

Table S4. Results from the conditional dependence Bayesian hierarchical latent (LC-BHSROC) model with weakly informative priors for the reference test.

| **Model-Conditional dependence** | **mean % (95%CI)** |
| --- | --- |
| ***5% of the maximum possible covariance*** |  |
| Sensitivity (Multiplex PCR) | 94,002 (92.13,95.67) |
| Specificity (Multiplex PCR) | 95.86 (94.5,97.2) |
| Sensitivity (culture) | 96.88 (95.04,98,79) |
| Specificity (culture) | 99,98 (99.96,1) |
| ***10% of the maximum possible covariance*** |  |
| Sensitivity (Multiplex PCR) | 93.9 (92.09,93.96) |
| Specificity (Multiplex PCR) | 95.95 (94.56,97.2) |
| Sensitivity (culture) | 96.66 (94.8,98,5) |
| Specificity (culture) | 99,98 (99.96,1) |
| ***20% of the maximum possible covariance*** |  |
| Sensitivity (Multiplex PCR) | 93.64 (91.82,95.34) |
| Specificity (Multiplex PCR) | 96.16 (94.89,97.39) |
| Sensitivity (culture) | 96.2 (94.32,98,02) |
| Specificity (culture) | 99,98 (99.96,1) |
| ***40% of the maximum possible covariance*** |  |
| Sensitivity (Multiplex PCR) | 92.95 (91.17,94.71) |
| Specificity (Multiplex PCR) | 96.67 (95.51,97.75) |
| Sensitivity (culture) | 94.94 (92.94,96.86) |
| Specificity (culture) | 99,99 (99.96,1) |
| ***50% of the maximum possible covariance*** |  |
| Sensitivity (Multiplex PCR) | 92.44 (90.61,94.21) |
| Specificity (Multiplex PCR) | 96.98 (95.89,97.96) |
| Sensitivity (culture) | 94.04 (91.97,96.16) |
| Specificity (culture) | 99,99 (99.96,1) |


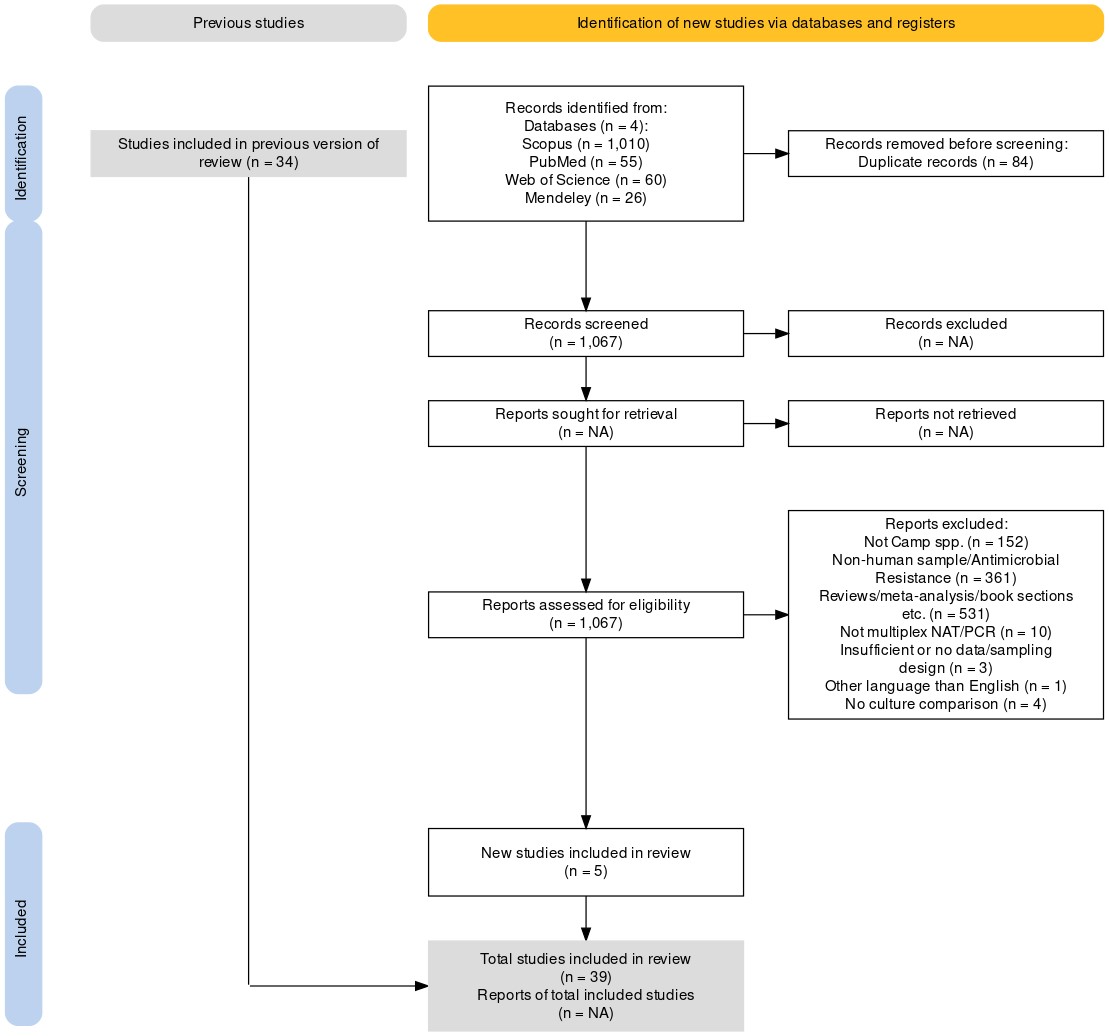


(40)

Figure S1: The PRISMA flow diagram of the systematic process for the selection of the relevant studies(40)

**1 Model code for rjags-** Bayesian hierarchical (BHSROC) model with or without a gold standard

model_jags <- "model

{ resdev <- sum(resdevi[]) # Residual deviance

for(i in 1:l) {

cell[i,1:4] ~ dmulti(cell_prob[i,1:4],n[i])

# Positive_Index - Positive_reference

cell_prob[i,1] <- pi[i]*( pp[i] * s2 ) + (1-pi[i])*( pn[i] * (1-c2))

# Positive_Index - Negative_reference

cell_prob[i,2] <- pi[i]*( pp[i] * (1-s2) ) + (1-pi[i])*( pn[i] * c2)

# Negative - Positive_reference

cell_prob[i,3] <- pi[i]*( (1-pp[i]) * s2 ) + (1-pi[i])*( (1-pn[i]) * (1-c2))

# Negative_Index - Negative_reference

cell_prob[i,4] <- pi[i]*( (1-pp[i]) * (1-s2) ) + (1-pi[i])*( (1-pn[i]) * c2)

### Conditional dependence:

#cell_prob[i,1] <- pi[i]*( pp[i] * s2 +covp[i] ) + (1-pi[i])*( pn[i] * (1-c2) +covn[i] )

#cell_prob[i,2] <- pi[i]*( pp[i] * (1-s2) -covp[i] ) + (1-pi[i])*( pn[i] * c2 -covn[i] )

#cell_prob[i,3] <- pi[i]*( (1-pp[i]) * s2 -covp[i] ) + (1-pi[i])*( (1-pn[i]) * (1-c2) -covn[i] )

# cell_prob[i,4] <- pi[i]*( (1-pp[i]) * (1-s2) +covp[i] ) + (1-pi[i])*( (1-pn[i]) * c2 +covn[i] )

# pp stands for : test under evaluation positive and reference standard positive (Crudely referred to true positive rate or sensitivity)

# pn stands for : test under evaluation positive and reference standard negative (Crudely referred to false positive rate or 1-specificity)

# Test under EVALUATION

logit(pp[i]) <- (theta[i] + 0.5*alpha[i])/exp(beta/2)

logit(pn[i]) <- (theta[i] - 0.5*alpha[i])*exp(beta/2)

#theta BETWEEN study variation of the cut-off values

theta[i] ~ dnorm(THETA,tau[1])

#alpha --> variation in the distribution of tp in each study

alpha[i] ~ dnorm(LAMBDA,tau[2])

# STUDY PREVALENCE PRIOR

pi[i] ~ dbeta(1,1)

# Fitted values

rhat[i,1] <- cell_prob[i,1]*n[i]

rhat[i,2] <- cell_prob[i,2]*n[i]

rhat[i,3] <- cell_prob[i,3]*n[i]

rhat[i,4] <- cell_prob[i,4]*n[i]

resdevi[i] <- 2*(cell[i,1]*log(cell[i,1]/rhat[i,1])+cell[i,2]*log(cell[i,2]/rhat[i,2])

+cell[i,3]*log(cell[i,3]/rhat[i,3])+cell[i,4]*log(cell[i,4]/rhat[i,4]))

# STUDY-LEVEL SENSITIVITY AND SPECIFICITY OF TEST UNDER EVALUATION

se[i] <- pp[i]

sp[i] <- 1-pn[i]

# limits of covariance parameters

#us[i]<-min(s2*(1-se[i]), (1-s2)*se[i])

#uc[i]<-min(c2*(1-sp[i]), (1-c2)*sp[i])

#ls[i]<- max(-(1-se[i])*(1-s2), -se[i]*s2)

#lc[i]<- max(-(1-sp[i])*(1-c2), -sp[i]*c2)

# prior distribution of transformed covariances on (0,1) range

#covp[i]~dunif(ls[i],us[i])

#covp[i]~dunif(0,us[i])

#covn[i]~dunif(lc[i],uc[i])

# covariance parameters as a fraction of their upper bound

#covp[i]<-0*us[i]

#covn[i]<-0*uc[i]

# replace 0 above by other percentages, e.g. 0.05, 0.1, 0.2, ..for conditional dependence.

}

# HYPER PRIOR DISTRIBUTIONS FOR PARAMETERS DEFINING TEST UNDER EVALUATION ACCURACY

THETA ~ dunif(-10,10)

LAMBDA ~ dunif(-6,6)

beta ~ dunif(-2,2)

for(j in 1:2) {

tau[j] <- pow(sigma[j],-2)

sigma[j] ~ dgamma(4,2)

}

###GOLD STANDARD:

s2<-1

c2<-1

###RESTRICTED PRIORS:

#s2 ~ dbeta(1,1)I(0.3,)

#c2 ~ dbeta(1,1)I(0.7,)

###INFORMATIVE PRIORS:

#s2 ~ dbeta(126.13,31.53)###min 0.66 max 0.90 mean 0.8

#s2 ~ dbeta(142.57,47.52)###min 0.6233 max 0.860 mean 0.75

#s2 ~ dbeta(3.71,1.59)###min 0.078 max 0.99948 mean 0.7

#s2 ~ dbeta(15.79,10.52)###min 0.2815 max 0,8884 mean 0.6

#c2 ~ dbeta(63.2,3.33) ###min 0.8 max 0,999 mean 0.9494

###NON INFORMATIVE PRIORS:

#s2 ~ dbeta(1,1)

#c2 ~ dbeta(1,1) #### OTHER PARAMETERS OF INTEREST

# Pooled sensitivity and specificity

Pooled_S<-1/(1+exp((-THETA-0.5*LAMBDA)/exp(beta/2)))

Pooled_C<-1/(1+exp((THETA-0.5*LAMBDA)*exp(beta/2)))

# Predicted sensitivity and specificity in a new study

theta_new ~ dnorm(THETA,tau[1])

alpha_new ~ dnorm(LAMBDA,tau[2])

S.new <- 1/(1+exp(-(theta_new+0.5*alpha_new)/exp(beta/2)))

C.new <- 1/(1+exp((theta_new-0.5*alpha_new)*exp(beta/2)))

#inits#LAMBDA,THETA,beta,s2,c2

#monitor deviance pD

}"

**2. Model code for rjags-**Bayesian bivariate with or without gold standard

model_jags <- "model

{

for(i in 1:l) {

cell[i,1:4] ~ dmulti(cell_prob[i,1:4],n[i])

# Positive_Index - Positive_reference

cell_prob[i,1] <- pi[i]*( se[i] * s2) + (1-pi[i])*( (1-sp[i])*(1-c2))

# Positive_Index - Negative_reference

cell_prob[i,2] <- pi[i]*( se[i] * (1-s2)) + (1-pi[i])*( (1-sp[i]) *c2)

# Negative - Positive_reference

cell_prob[i,3] <- pi[i]*( (1-se[i]) * s2 ) + (1-pi[i])*( sp[i]*(1-c2))

# Negative_Index - Negative_reference

cell_prob[i,4] <- pi[i]*( (1-se[i]) * (1-s2)) + (1-pi[i])*( sp[i]*c2)

# Hierarchical prior for multiplex PCR

logit(se[i]) <- l1[i,1]

logit(sp[i]) <- l1[i,2]

l1[i,1:2] ~ dmnorm(mu[], T[,])

# Prior distribution on prevalence

pi[i] ~ dbeta(1,1)

}

# Hyper priors for multiplex

mu[1] ~ dnorm(0,0.25)

mu[2] ~ dnorm(0,0.25)

T[1:2,1:2]<-inverse(TAU[1:2,1:2])

#### BETWEEN-STUDY VARIANCE-COVARIANCE MATRIX

TAU[1,1] <- tau[1]*tau[1]

TAU[2,2] <- tau[2]*tau[2]

TAU[1,2] <- rho*tau[1]*tau[2]

TAU[2,1] <- rho*tau[1]*tau[2]

#### prec = between-study precision in the logit(sensitivity) and logit(specificity)

prec[1] ~ dgamma (2,0.5)

prec[2] ~ dgamma(2,0.5)

rho ~ dunif(-1,1)

#### BETWEEN_STUDY STANDARD DEVIATION IN THE LOGIT(SENSITIVITY) AND LOGIT(SPECIFICITY)

tau[1]<-pow(prec[1],-0.5)

tau[2]<-pow(prec[2],-0.5)

#### BETWEEN_STUDY VARIANCE IN THE LOGIT(SENSITIVITY) AND LOGIT(SPECIFICITY)

tau.sq[1] <- pow(tau[1], 2)

tau.sq[2] <- pow(tau[2], 2)

###GOLD STANDARD:

s2<-1

c2<-1

###RESTRICTED PRIORS:

#s2 ~ dbeta(1,1)I(0.3,)

#c2 ~ dbeta(1,1)I(0.7,)

###INFORMATIVE PRIORS:

#s2 ~ dbeta(126.13,31.53)###min 0.66 max 0.90 mean 0.8

#s2 ~ dbeta(142.57,47.52)###min 0.6233 max 0.860 mean 0.75

#s2 ~ dbeta(3.71,1.59)###min 0.078 max 0.99948 mean 0.7

#s2 ~ dbeta(15.79,10.52)###min 0.2815 max 0,8884 mean 0.6

#c2 ~ dbeta(63.2,3.33) ###min 0.8 max 0,999 mean 0.9494

###NON INFORMATIVE PRIORS:

#s2 ~ dbeta(1,1)

#c2 ~ dbeta(1,1)

#### SUMMARY SENSITIVITY AND SPECIFICITY

Pooled_S<-1/(1+exp(-mu[1]))

Pooled_C<-1/(1+exp(-mu[2]))

#### PREDICTED SENSITIVITY AND SPECIFICITY OF INDEX TEST IN A FUTURE STUDY

l.new[1:2] ~ dmnorm(mu[],T[,])

S_new <- 1/(1+exp(-l.new[1]))

C_new <- 1/(1+exp(-l.new[2]))

#inits#

#monitor deviance pD

}"

References:

1. Martín A, Pérez-Ayala A, Chaves F, Lora D, Orellana MÁÁ. Evaluation of the multiplex PCR Allplex-GI assay in the detection of bacterial pathogens in diarrheic stool samples. Journal of Microbiological Methods . 2018 Jan;144:33–6. Available from: https://linkinghub.elsevier.com/retrieve/pii/S0167701217302890

2. Jo SJ, Kang HM, Kim JO, Cho H, Heo W, Yoo IY, et al. Evaluation of the biofire gastrointestinal panel to detect diarrheal pathogens in pediatric patients. Diagnostics [Internet]. 2022 Dec 24;12(1):34. Available from: https://www.mdpi.com/2075-4418/12/1/34

3. Rintala A, Munukka E, Weintraub A, Ullberg M, Eerola E. Evaluation of a multiplex real-time PCR kit Amplidiag® Bacterial GE in the detection of bacterial pathogens from stool samples. Journal of microbiological methods . 2016 Sep;128:61–5. Available from: https://linkinghub.elsevier.com/retrieve/pii/S0167701216301816

4. Koo SH, Heng YX, Jiang B, Ng LSY, Sim DMF, Tan TY. Evaluation of the clinical sensitivity and specificity of the BD MaxTM Enteric Bacterial Panel for molecular detection of pathogens for acute gastroenteritis in the Singaporean population. JOURNAL OF MICROBIOLOGICAL METHODS . 2022 Jun 1 [cited 2022 Sep 8];197:106478. Available from: https://linkinghub.elsevier.com/retrieve/pii/S0167701222000732

5. Ozcan N, Bacalan F, Cakir F, Bilden A, Genisel N, Dal T, et al. Culture and culture-independent diagnostic tests in Campylobacter enteritis. JOURNAL OF INFECTION IN DEVELOPING COUNTRIES . 2022 Apr;16(4):616–21. Available from: https://www.scopus.com/inward/record.uri?eid=2-s2.0-85129909250&doi=10.3855%2Fjidc.14902&partnerID=40&md5=bc4228365905e63a738b726896cbc901

6. Berenger, B. M., Chui, L., Ferrato, C., Lloyd, T., Li, V., & Pillai DR. Performance of four commercial real-time PCR assays for the detection of bacterial enteric pathogens in clinical samples. International Journal of Infectious Diseases . 2021 Jan 1 [cited 2022 Sep 8];114:195–201. Available from: https://www.sciencedirect.com/science/article/pii/S1201971221008316

7. Wohlwend N, Tiermann S, Risch L, Risch M, Bodmer T, Bodmer labormedizinisches zentrum Risch T, et al. Evaluation of a Multiplex Real-Time PCR Assay for Detecting Major Bacterial Enteric Pathogens in Fecal Specimens: Intestinal Inflammation and Bacterial Load Are Correlated in Campylobacter Infections. JOURNAL OF CLINICAL MICROBIOLOGY. 2016 Sep;54(9):2262–6.

8. Harrington SM, Buchan BW, Doern C, Fader R, Ferraro MJ, Pillai DR, et al. Multicenter Evaluation of the BD Max Enteric Bacterial Panel PCR Assay for Rapid Detection of Salmonella spp., Shigella spp., Campylobacter spp. (C. jejuni and C. coli), and Shiga Toxin 1 and 2 Genes. Gilligan PH, editor. Journal of Clinical Microbiology [Internet]. 2015 May [cited 2021 Apr 3];53(5):1639–47. Available from: https://jcm.asm.org/content/53/5/1639

9. Knabl L, Grutsch I, Orth-Höller D. Comparison of the BD MAX® Enteric Bacterial Panel assay with conventional diagnostic procedures in diarrheal stool samples. European Journal of Clinical Microbiology & Infectious Diseases [Internet]. 2016 Jan 13;35(1):131–6. Available from: http://link.springer.com/10.1007/s10096-015-2517-4

10. McAuliffe G, Bissessor L, Williamson D, Moore S, Wilson J, Dufour M, et al. Use of the EntericBio Gastro Panel II in a diagnostic microbiology laboratory: challenges and opportunities. PATHOLOGY. 2017 Jun;49(4):419–22. Available from: https://linkinghub.elsevier.com/retrieve/pii/S0031302516405581

11. O’Leary J, Corcoran D, Lucey B. Comparison of the EntericBio Multiplex PCR System with Routine Culture for Detection of Bacterial Enteric Pathogens. JOURNAL OF CLINICAL MICROBIOLOGY. 2009 Nov [cited 2021 Apr 25];47(11):3449–53. Available from: http://jcm.asm.org/

12. McAuliffe GN, Anderson TP, Stevens M, Adams J, Coleman R, Mahagamasekera P, et al. Systematic application of multiplex PCR enhances the detection of bacteria, parasites, and viruses in stool samples. Journal of Infection [Internet]. 2013 Aug;67(2):122–9. Available from: https://linkinghub.elsevier.com/retrieve/pii/S0163445313000984

13. Ghoshal U, Tejan N, Sisodia J, Verma S, Prasad N, UC G. The utility of multiplex polymerase chain reaction for diagnosis of infectious diarrhoea in a tropical country. Indian Journal of Medical Microbiology. 2021 Jul 1;39(3):323–7.

14. Buss SN, Leber A, Chapin K, Fey PD, Bankowski MJ, Jones MK, et al. Multicenter Evaluation of the BioFire FilmArray Gastrointestinal Panel for Etiologic Diagnosis of Infectious Gastroenteritis. Burnham C-AD, editor. Journal of Clinical Microbiology. 2015 Mar;53(3):915–25. Available from: https://jcm.asm.org/content/53/3/915

15. Cybulski RJ, Bateman AC, Bourassa L, Bryan A, Beail B, Matsumoto J, et al. Clinical Impact of a Multiplex Gastrointestinal Polymerase Chain Reaction Panel in Patients With Acute Gastroenteritis. Clinical Infectious Diseases [Internet]. 2018 Apr 25;67(11):1697–704. Available from: https://academic.oup.com/cid/advance-article/doi/10.1093/cid/ciy357/4985150

16. Huang RSP, Johnson LC, Pritchard L, Hepler R, Ton TT, Dunn JJ. Performance of the Verigene® enteric pathogens test, Biofire FilmArray^TM^ gastrointestinal panel and Luminex xTAG® gastrointestinal pathogen panel for detection of common enteric pathogens. Diagnostic Microbiology and Infectious Disease. 2016 Dec 1;86(4):336–9.

17. Kutsuna S, Hayakawa K, Mezaki K, Yamamoto K, Ohmagari N. Spectrum of enteropathogens in cases of traveler’s diarrhea that were detected using the FilmArray GI panel: New epidemiology in Japan. Journal of Infection and Chemotherapy . 2021 Jan 1 [cited 2021 Mar 18];27(1):49–54. Available from: https://www.sciencedirect.com/science/article/pii/S1341321X20302890

18. Wiemer D, Loderstaedt U, von Wulffen H, Priesnitz S, Fischer M, Tannich E, et al. Real-time multiplex PCR for simultaneous detection of Campylobacter jejuni, Salmonella, Shigella and Yersinia species in fecal samples. International Journal of Medical Microbiology. 2011 Nov;301(7):577–84. Available from: https://linkinghub.elsevier.com/retrieve/pii/S1438422111000658

19. Zaghloul MZ, Farouk N, Galal ZA. Detection of cambylobacter spp. in stool samples by new methods in comparison to culture. Life Science Journal-ACTA Zhengzhou university Overseas edition [Internet]. 2012 Dec 25 [cited 2021 Mar 28];9(4):2566–71. Available from: https://www.researchgate.net/publication/288103295_Detection_of_cambylobacter_spp_in_stool_samples_by_new_methods_in_comparison_to_culture

20. Hu Q, Lyu DY, Shi X, Jiang Y, Lin Y, Li Y, et al. A Modified Molecular Beacons–Based Multiplex Real-Time PCR Assay for Simultaneous Detection of Eight Foodborne Pathogens in a Single Reaction and Its Application. Foodborne Pathogens and Disease. 2014 Mar 1;11(3):207–14. Available from: http://www.liebertpub.com/doi/10.1089/fpd.2013.1607

21. Kawase J, Etoh Y, Ikeda T, Yamaguchi K, Watahiki M, Shima T, et al. An Improved Multiplex Real-Time SYBR Green PCR Assay for Analysis of 24 Target Genes from 16 Bacterial Species in Fecal DNA Samples from Patients with Foodborne Illnesses. Japanese Journal of Infectious Diseases. 2016 May [cited 2021 Feb 10];69(3):191–201. Available from: https://www.jstage.jst.go.jp/article/yoken/69/3/69_JJID.2015.027/_article

22. Kellner T, Parsons B, Chui L, Berenger BMBM, Xie J, Burnham C-ADC-AD, et al. Comparative Evaluation of Enteric Bacterial Culture and a Molecular Multiplex Syndromic Panel in Children with Acute Gastroenteritis. Ledeboer NA, editor. Journal of Clinical Microbiology 2019 Apr 10;57(6). Available from: https://jcm.asm.org/content/57/6/e00205-19

23. Navidad JF, Griswold DJ, Gradus MS, Bhattacharyya S. Evaluation of Luminex xTAG Gastrointestinal Pathogen Analyte-Specific Reagents for High-Throughput, Simultaneous Detection of Bacteria, Viruses, and Parasites of Clinical and Public Health Importance. JOURNAL OF CLINICAL MICROBIOLOGY. 2013 Sep;51(9):3018–24.

24. Deng J, Luo X, Wang R, Jiang L, Ding X, Hao W, et al. A comparison of Luminex xTAG® Gastrointestinal Pathogen Panel (xTAG GPP) and routine tests for the detection of enteropathogens circulating in Southern China. Diagnostic Microbiology and Infectious Disease. 2015 Nov 1 [cited 2021 Feb 10];83(3):325–30. Available from: https://linkinghub.elsevier.com/retrieve/pii/S0732889315002886

25. Duong VT, Phat VV, Tuyen HT, Dung TTN, Trung PD, Minh P Van, et al. Evaluation of Luminex xTAG Gastrointestinal Pathogen Panel Assay for Detection of Multiple Diarrheal Pathogens in Fecal Samples in Vietnam. Richter SS, editor. JOURNAL OF CLINICAL MICROBIOLOGY. 2016 Apr;54(4):1094–100. Available from: https://jcm.asm.org/content/54/4/1094

26. Halligan E, Edgeworth J, Bisnauthsing K, Bible J, Cliff P, Aarons E, et al. Multiplex molecular testing for management of infectious gastroenteritis in a hospital setting: a comparative diagnostic and clinical utility study. Clinical Microbiology and Infection. 2014 Aug [cited 2021 Jan 25];20(8):O460–7. Available from: https://linkinghub.elsevier.com/retrieve/pii/S1198743X14603451

27. Tilmanne A, Martiny D, Quach C, Wautier M, Vandenberg O, Lepage P, et al. Enteropathogens in paediatric gastroenteritis: comparison of routine diagnostic and molecular methods. Clinical Microbiology and Infection. 2020 Dec 1 [cited 2021 Jan 25];25(12):1519–24. Available from: https://doi.org/10.1016/j.cmi.2019.07.021

28. Claas. EC, Burnham. C-AD, Tony Mazzulli, Kate Templeton and FT. Performance of the xTAG Gastrointestinal Pathogen Panel, a Multiplex Molecular Assay for Simultaneous Detection of Bacterial, Viral, and Parasitic Causes of Infectious Gastroenteritis. Journal of Microbiology and Biotechnology [Internet]. 2013 Jul 28;23(7):1041–5. Available from: http://www.jmb.or.kr/journal/viewJournal.html?year=2013&vol=23&num=7&page=1041

29. Huang SH, Lin YF, Tsai MH, Yang S, Liao ML, Chao SW, et al. Detection of common diarrhea-causing pathogens in Northern Taiwan by multiplex polymerase chain reaction. Medicine (United States). 2018 Jun 1;97(23).

30. Pankhurst L, Macfarlane-Smith L, Buchanan J, Anson L, Davies K, O’Connor L, et al. Can rapid integrated polymerase chain reaction-based diagnostics for gastrointestinal pathogens improve routine hospital infection control practice? A diagnostic study. Health Technology Assessment. 2014 Aug;18(53):1–167. Available from: https://www.journalslibrary.nihr.ac.uk/hta/hta18530/

31. Patel A, Navidad J, Bhattacharyya S. Site-specific clinical evaluation of the Luminex xTAG gastrointestinal pathogen panel for detection of infectious gastroenteritis in fecal specimens. Journal of Clinical Microbiology. 2014;52(8):3068–71.

32. Perry MD, Corden SA, Howe RA. Evaluation of the luminex xTAG Gastrointestinal Pathogen Panel and the Savyon Diagnostics Gastrointestinal Infection Panel for the detection of enteric pathogens in clinical samples. Journal of Medical Microbiology. 2014 Nov 1;63(11):1419–26.

33. Dror Ken, Pavlotzky E, Barak M, Dror SK, Pavlotzky E, Barak M, et al. Evaluation of the NanoCHIP® Gastrointestinal Panel (GIP) Test for Simultaneous Detection of Parasitic and Bacterial Enteric Pathogens in Fecal Specimens. Chang Y-F, editor. PloS one. 2016 Jul 22;11(7):e0159440. Available from: https://dx.plos.org/10.1371/journal.pone.0159440

34. Roy C, Robert D, Bénéjat L, Buissonnière A, Ducournau A, Mégraud F, et al. Performance evaluation of the Novodiag® Bacterial GE+ multiplex PCR assay. Onderdonk AB, editor. Journal of Clinical Microbiology. 2020 [cited 2021 Mar 28];58(10). Available from: https://jcm.asm.org/content/58/10/e01033-20

35. Buchan BW, Olson WJ, Pezewski M, Marcon MJ, Novicki T, Uphoff TS, et al. Clinical evaluation of a real-time PCR assay for identification of salmonella, shigella, campylobacter (campylobacter jejuni and C. coli), and shiga toxin-producing escherichia coli isolates in stool specimens. Journal of Clinical Microbiology [Internet]. 2013 Dec 1 [cited 2021 Feb 27];51(12):4001–7. Available from: https://jcm.asm.org/content/51/12/4001

36. Coupland L.J MI, Meader E, Cowley K, Alcock L, Naunton J, Gr DJ, et al. Simultaneous detection of viral and bacterial enteric pathogens using the Seeplex 1 Diarrhea ACE detection system. 2012 [cited 2021 Apr 3]; Available from: https://doi.org/10.1017/S0950268812002622

37. Bessède E, Delcamp A, Sifré E, Buissonnière A, Mégraud F. New Methods for Detection of Campylobacters in Stool Samples in Comparison to Culture. Journal of Clinical Microbiology. 2011 Mar [cited 2021 Feb 3];49(3):941–4. Available from: http://www.ncbi.nlm.nih.gov/pubmed/21209172

38. Onori M, Coltella L, Mancinelli L, Argentieri M, Menichella D, Villani A, et al. Evaluation of a multiplex PCR assay for simultaneous detection of bacterial and viral enteropathogens in stool samples of paediatric patients. Diagnostic Microbiology and Infectious Disease. 2014;79(2):149–54.

39. Kosai K, Suzuki H, Tamai K, Okada Y, Akamatsu N, Ueda A, et al. Multicenter evaluation of Verigene Enteric Pathogens Nucleic Acid Test for detection of gastrointestinal pathogens. Scientific Reports. 2021 Feb 4 [cited 2022 Sep 8];11(1):3033. Available from: http://www.nature.com/articles/s41598-021-82490-z

40. Haddaway NR, Page MJ, Pritchard CC, McGuinness LA. PRISMA2020: An R package and Shiny app for producing PRISMA 2020-compliant flow diagrams, with interactivity for optimised digital transparency and Open Synthesis. Campbell Systematic Reviews. 2022 Jun 1;18(2):e1230. Available from: https://doi.org/10.1002/cl2.1230
